# Recent Trends in Malaria Vaccine Research Globally: A Bibliometric Analysis From 2005 to 2022

**DOI:** 10.1155/2024/8201097

**Published:** 2024-10-24

**Authors:** Muhammad Chutiyami

**Affiliations:** School of Nursing and Midwifery, Faculty of Health, University of Technology Sydney, Sydney, Australia

**Keywords:** bibliometric analysis, citations, malaria vaccine, *Plasmodium*

## Abstract

**Aim:** Malaria vaccine is one of the critical areas in tropical health research, considering the success recorded in other vaccine-preventable diseases. This study is aimed at reviewing recent trends in global malaria vaccine research from 2005 to 2022.

**Method:** A validated search strategy was undertaken to identify scientific literature on the malaria vaccine in the Scopus database. Bibliometric indicators identified include a pattern of publication growth and citations over the study period; top authors, countries, funding organizations, and journals; keywords, including different malarial parasite species, and the overall research themes.

**Result:** A total of 6457 documents were found from 2005 to 2022, published in 160 journals/sources in 189 countries/territories. Malaria Journal published the highest number of research outputs (478, 7.4%) within the study period, and the highest number of documents (468, 7.3%) were published in 2021. There were 214,323 total citations, with 33.2 average citations per document and 167 documents' h-index. The United States, United Kingdom, and Australia combined produced more than 60% of the publication output, with most collaboration with African countries such as Kenya. *Plasmodium falciparum* is the most occurring parasite species keyword (754, 11.7%), with a growing interest in *Plasmodium knowlesi* (30, 0.5%). Merozoite surface protein, characterization, trials, infant/children, traveler, and research/review were the six themes that emerged from the studies.

**Conclusion:** The last one and half decades have seen a significant increase in malaria vaccine research and citations, mainly targeting vaccine development, safety, and efficacy in Africa. This necessitates more international efforts to improve the vaccines' effectiveness considering different *Plasmodium* species.

## 1. Introduction

In recent years, the malaria vaccine has been one of the critical areas in public health research, considering the success recorded in other vaccine-preventable diseases [1, 2]. Malaria is a life-threatening disease caused by various *Plasmodium* (*P.*) parasite species: *P. falciparum*, *P. vivax*, *P. malariae*, *P. ovale*, or *P. knowlesi*. The two species, *P. falciparum* and *P. vivax*, were reported to contribute to the higher burden of malaria morbidities and mortalities [3]. The World Health Organization (WHO) 2023 malaria key facts indicate an estimated prevalence of 249 million malaria cases and 608,000 deaths in 85 endemic countries [3]. The WHO African region carries a disproportionately high burden, accounting for 94% of cases and 95% of deaths, with children under five the most vulnerable group [3]. Control measures such as the use of long-lasting insecticide-treated nets and artemisinin combination therapy have been promising in reducing the incidence of malaria [4]; however, these methods were not sustainable, particularly in malaria-endemic countries of Africa, necessitating the need for long-lasting solutions such as vaccines. Our recent review indicates the willingness of African communities to accept the malaria vaccine, considering the efficacy and side effects [5].

Recent bibliometric research indicated an increasing number of malaria research from the 1970s [6], corresponding to malaria vaccines' historical development. Traditionally, approaches to developing subunit vaccines target specific parasite antigens; however, the efficacy of these vaccines decreases quickly over time [7]. As a result, scientists focus on exploring noble strategies such as RNA-based vaccines, viral-vectored vaccines as well as whole parasite vaccines [8–10]. The RNA-based vaccine approach uses messenger RNA (mRNA) to stimulate body cells to produce specific malaria antigens [8]. mRNA malaria vaccines, including those developed by the malaria vaccine initiative (MVI), show promising results in preclinical and early clinical trials [11]. Conversely, viral-vectored vaccines use modified viruses that are genetically altered to induce malaria antigens into the body [10]. The adenovirus and poxvirus vectors, which stimulate cellular and antibody-mediated immune responses, are commonly employed in this strategy [10]. On the other hand, the whole parasite vaccine takes the approach of the natural infection mechanism in the body, which induces stronger immunity [9]. It involves attenuated/weakened malaria parasites to generate a more comprehensive immune response. Clinical trials of such vaccines, like the PfSPZ (*Plasmodium falciparum sporozoites*), have shown an initial positive outcome in providing significant protection against malaria infection [12], although wide-scale trials are still required.

Collaboration between malaria-endemic countries and international partners has been promising, with several vaccines being developed in Western countries and undergoing clinical trials among the African population. This approach has been, in particular, the case in the most researched malaria vaccine, RTS, S, over three decades [13, 14], as well as more recent and effective vaccines like the R21 [15]. A new trend has shown that a combination of RTS, S (*repeat T-cell epitope surface antigen with additional surface antigen*) with chemoprophylaxis as opposed to the vaccine alone [16, 17], as well as R21 vaccine before seasonal malaria [18], yields better results. This could be associated with the complex life cycle of malarial parasites, requiring a multifaceted approach.

Given the diverse research in this area, this review sought to look into the recent trends in global malaria vaccine research through a bibliometric analysis. The study is aimed at building on a previous bibliometric analysis of global malaria vaccine research conducted in 2005, covering research published from 1972 to 2004 [19]. The outcome of this review will contribute to shaping policies, provide new directions for research, and bring more awareness to malaria vaccine research. As research progresses, international collaboration, suitable investment, and government and community efforts will be required to contain malaria in the near future.

## 2. Methods

### 2.1. Research Design

This study adopted a bibliometric analysis of published research studies from 2005 to 2022. As an emerging field in scientific research, bibliometric analysis enables researchers to systematically explore, collect, and analyze large volumes of scientific data to identify key landmarks and emerging research trends in a specific field [20]. Oliveira et al., [21] added that bibliometric analysis offers insight into the performance of authors, articles, journals, institutions, and countries. This study adheres to key performance indicators and science mapping outlined by Donthu et al. [20], presenting a systematic bibliometric analysis guideline.

### 2.2. Database

The Scopus database was adopted for this study to identify the existing body of literature on malaria vaccine research. Scopus has been used for bibliometric analysis in various bibliometric studies, including general vaccine hesitancy [22], COVID-19 vaccine research [23], and climate change [24], due to its rich resource that makes it a reliable source of bibliometric data. Additionally, the Scopus database has been demonstrated to have a larger collection of reliable scientific literature than other reputable databases [25, 26].

### 2.3. Search Strategy and Screening

The search strategy for this review was carefully developed through a trial of different scenarios to address variations of the key terms “malaria” and “vaccine” to avoid false positive and false negative outcomes. Following the trials, the following search query was used in the Scopus advanced search interface: (TITLE-ABS-KEY (malaria OR *Plasmodium OR falciparum OR* “*Plasmodium falciparum*” *OR* “*P. falciparum*” *OR* “*Plasmodium malariae*” *OR* “*P. malariae*” *OR* “*Plasmodium vivax*” *OR* “*P. vivax*” *OR* “*Plasmodium ovale*” *OR* “*P. ovale*” *OR* “*Plasmodium knowlesi*” *OR* “*P. knowlesi*”) AND TITLE-ABS-KEY (vaccin⁣^∗^ OR “autogenous vaccine” OR autovaccine)) AND PUBYEAR >2004 AND PUBYEAR <2023, of which the first hit resulted in 10,931 documents.

A validation screening was undertaken to identify false positive results by examining the first 100 documents in line with previous bibliometric analysis [22]. This resulted in identifying nine editorial reports with authors' keywords (i.e., research expertise/interest) containing the search terms rather than keywords of the documents. The search was then modified to title and abstract by excluding the keywords, resulting in 7229 documents.

A further examination of the “document types” under Scopus limiters was conducted to identify only scientific research papers. This was done by limiting the search outcome to the individual document type (article, review, note, book chapter, short survey, conference paper, editorial, and letter) at a time and then reviewing the title/abstract of the first 10 documents for relevance. Through this examination, only three document types were found to be scientific research papers that met the aim of the study. The others were commentaries/notes of another research and one retracted paper. Therefore, the final search was narrowed to the three document types: articles, reviews, and short surveys. A further examination of the first 100 overall results reveals no false positives, resulting in 6457 documents included in the analysis.

### 2.4. Bibliometric Indicators and Analysis

Scopus features “analyze result,” and Microsoft Excel and VOS viewer software were used to examine the bibliometric indicators of the search results. Percentages and frequencies were used to estimate each item and presented on tables, charts, and maps as appropriate.

In Scopus, overall indicators were computed, including h-index, documents by year of publication, source of data, authors/authors' affiliations, country/territory of research, and source of funding. The data was exported as a CSV (comma-separated values) file to Microsoft Excel, where the yearly citation report and the journal rankings were computed.

Visualization of the most frequent items was limited to the Top 10 in line with a prior study [22], including authors, countries, funding organizations, and journals. The Top 10 authors' statistics were computed by calculating the number of times an author's name appeared in the literature, irrespective of the author's position. Similarly, the Top 10 countries were computed based on the country of affiliation of each author, regardless of the author's position in the article.

The VOSviewer used data to establish a network of key indicators, including coauthorship between countries, authors and index keywords co-occurrence, and research topic themes. The keywords analysis identified occurrences of different malarial parasite species through the VOSviewer. To ensure better visibility in the VOSviewer, the network was narrowed to a minimum of five occurrences (recommended threshold) for each indicator. Furthermore, the number of clusters of items and the total number of links were reported alongside the results of the VOSviewer output.

## 3. Results

### 3.1. Pattern of Publication Output and Citation Reports

A total of 6457 relevant documents were identified, including 122 (1.9%) short surveys, 1296 (20.1%) review papers, and 5039 (78.0%) primary research articles. The majority of the articles (95.7%) were in English, while the others were in French (1.3%), German (1.0%), Chinese (0.8%), and other languages (1.2%).

Table 1 details the breakdown of the 6457 documents by year and their citation count. The peak in number of papers published (7.3%) was in 2021, followed by 2022, each with over 400 documents. Conversely, the highest citation count per document was published in 2010 compared to the lowest in 2022, out of the 214,323 total citations. The overall average citation per document was 33.2, ranging from 4.2 to 58.6. The h-index of the 6457 article was 176; the top cited article throughout the study period was an experimental study published in “*Nature*” in 2006, which received 2255 citations [27]. Within the last decade, the top cited article was a clinical trial on RTS, S vaccine published by “*The Lancet*” in 2015, with 915 total citations [28].

### 3.2. Overview of Top 10 Authors, Countries, Funding Organizations, and Journals

**Figure 1:**
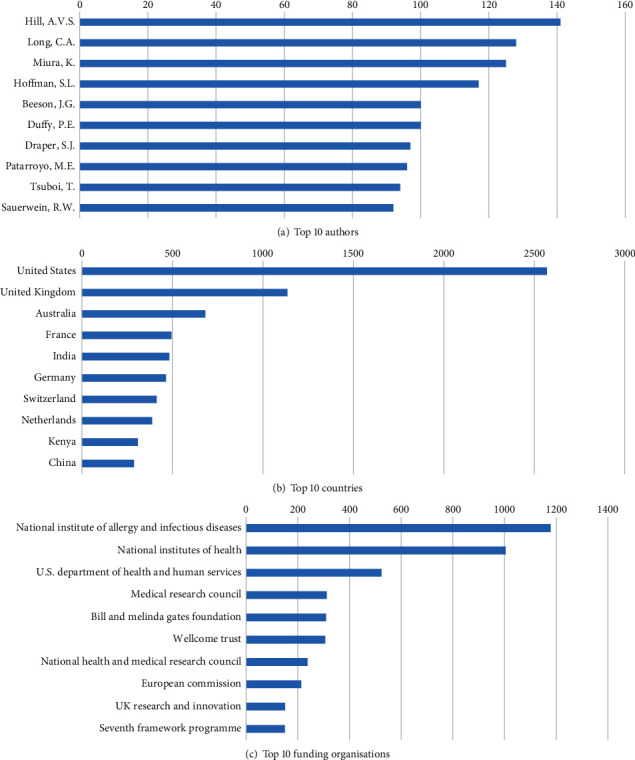
Malaria vaccine research Top 10 authors, countries, and funding organizations from 2005 to 2022.

The Top 10 authors have a total of 1090 (17.0%) documents, of which Hill, A.V.S. from the Jenner Institute, United Kingdom, had the highest publication output (Figure 1(a)). The United States produced more than 1/3 of all the publications' output, and with the following 2 countries (the United Kingdom and Australia), 68.0% of the research outputs (Figure 1(b)). Of the Top 10 countries, two were from Asia (China and India) and one from Africa (Kenya), while the remaining were in Europe, America and Australia. Similarly, the US National Institute of Allergy and Infectious Diseases funded the highest number of research output (18%). In comparison, the European Union Seventh Framework Programme funded the least number of documents (2.3%) among the Top 10 funding organizations (Figure 1(c)).

**Figure 2:**
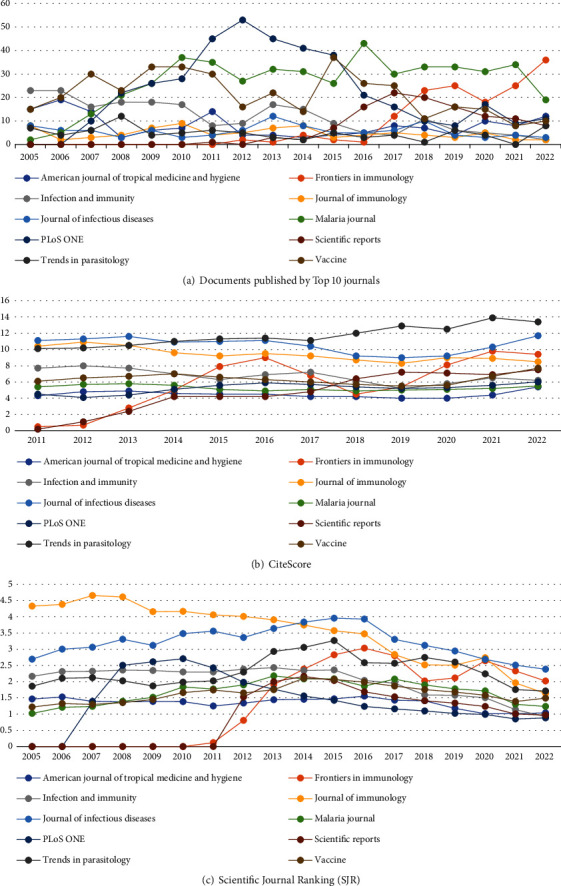
Malaria vaccine research Top 10 journals, CiteScore, and Scientific Journal Rankings from 2005 to 2022.

A total of 160 journals/sources published the 6457 documents identified, out of which the Top 10 had a total publication output of 2153 (33.3%). Malaria Journal, an open-access journal from Springer Nature, had the highest total output, 478 (7.4%). However, PLOS One journal had the highest number of documents from 2011 to 2015 compared to the other journals.  Figure 2(a) shows the distribution of the documents by year across the Top 10 journals. The journals have different CiteScore (number of citations to documents) across the years, with the data available from 2011 to 2022 (Figure 2(b)). Most journals maintained a similar CiteScore, except Scientific Reports and Frontiers in Immunology (open access), which changed significantly from 0.2 and 0.5 in 2011 to 7.5 and 9.4, respectively, in 2022. Like the CiteScore, the Scientific Journal Ranking (SJR) of the Top 10 journals varies (Figure 2(c)). Between 2005 and 2012, the Journal of Immunology had the highest SJR, while the Journal of Infectious Disease scored the highest (2.386) in 2022. PLOS One had one of the most considerable variations, with an initial SJR score of 0 in 2006 and a peak of 2.7 in 2010, which reduced to 0.885 in 2022.

### 3.3. Coauthorship Between Countries

**Figure 3:**
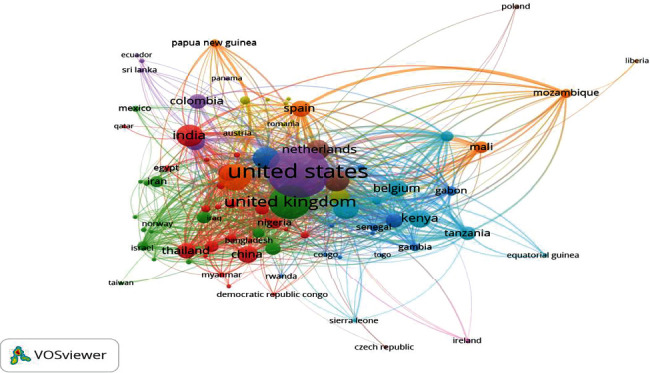
Malaria vaccine research coauthorship between countries (91 countries/territories, 9 clusters, and 1555 links from VOSviewer).

A total of 189 countries/territories authored the published documents; 91 have at least five papers (VOSviewer recommended threshold for visualization) coauthored with another country. The coauthorship links between the countries are shown in Figure 3. It can be seen from the figure that the United States and the United Kingdom dominate the majority of the authorship; however, the links indicating coauthorships were primarily with African countries such as Kenya, Tanzania, and Mozambique. Countries in other regions with a sizeable coauthorship network include India and Papua New Guinea.

### 3.4. Co-Occurrence of Keywords (Index and Author's Keywords)

**Figure 4:**
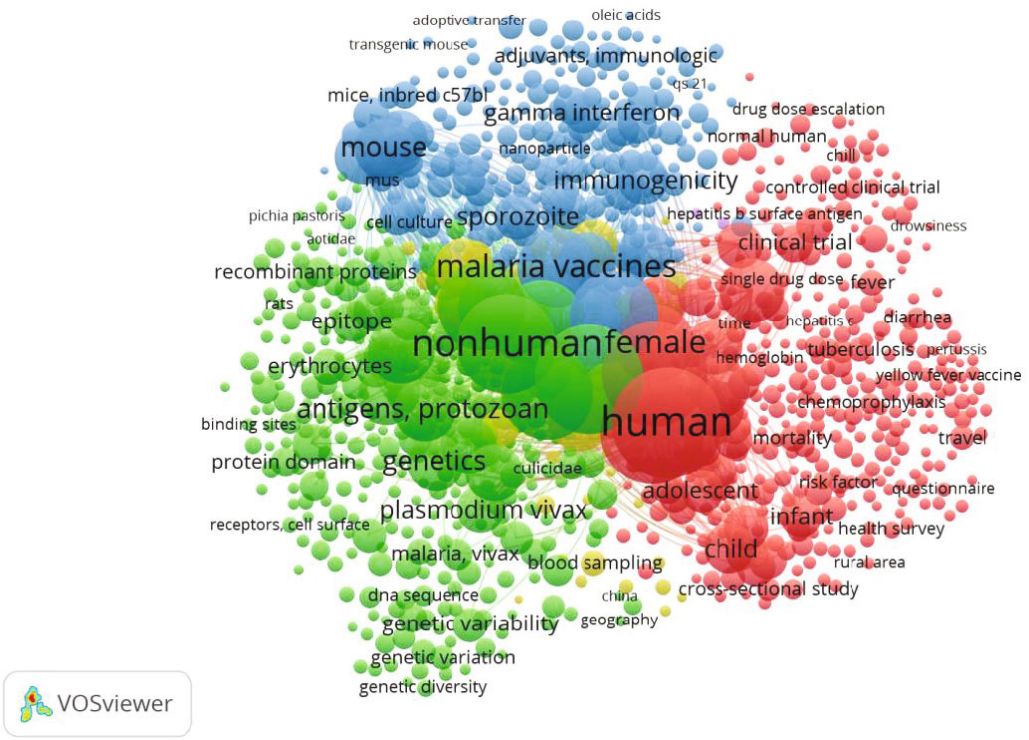
Malaria vaccine research co-occurrence of keywords (Top 1000 keywords, 5 clusters, and 29,1420 links from VOSviewer).

A total of 26,114 keywords (author and index) across the documents, with 4838 keywords occurring at least five times (threshold), were used for this analysis. The total co-occurrence between the different keywords is displayed in Figure 4. Two of the most occurring keywords, “human” and “animals,” signify the major domains of the vaccine research, largely co-occurring with the subjects “child” and “mouse.” Other important links include vaccination, genetics, immunogenicity, and drug efficacy. Similar patterns appeared when narrowed to the author's (Figure S1) and index (Figure S2) keywords, but in addition, associated infectious diseases, such as COVID-19, tuberculosis, hepatitis, dengue, typhoid, cholera, and rabies, were identified.

A further examination of the authors' keywords (Figure S1) was conducted to identify the occurrence of different malaria species among the 6457 documents that specify the species; these include 754 *P. falciparum*, 336 *P. vivax*, 71 *P. berghei*, 40 *P. yoelii*, 30 *P. knowlesi*, 16 *P. chabaudi*, 6 *P. cynomolgi*, and 5 *P. ovale* appeared, respectively. Accordingly, the most occurring species, *P. falciparum*, is connected in 3 clusters, with malaria, var2csa protein, and pfs25 protein, while the least, *P. ovale* was connected to 1 cluster, atovaquone.

### 3.5. Research Themes Through a Network of the Most Occurring Terms in Documents' Title/Abstract

**Figure 5:**
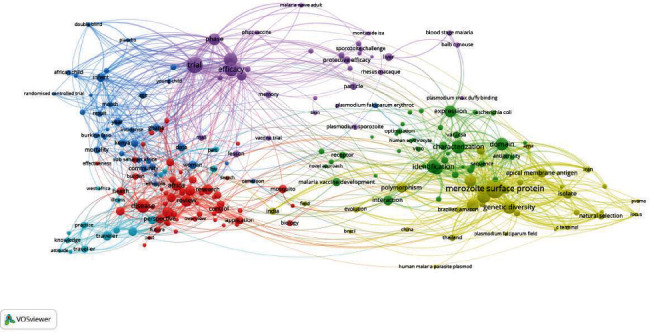
Malaria vaccine research themes through the document's title/abstract (367 terms, 6 clusters, and 2644 links from VOSviewer).

A total of 13889 specific terms exist in the documents' title/abstract, out of which 367 terms occurred at least five times (threshold). Six clusters representing six research themes were identified (Figure 5). Density visualization of these research themes was presented (Figure S3).

Of the six research themes (Figure 5), Theme 1 (yellow-green cluster) and Theme 2 (Kelly-green cluster) represent malaria vaccine design/development. Theme 1 mainly focuses on merozoite surface protein (MSP-1, the most abundant protein on the surface of the erythrocyte-invading *Plasmodium* merozoite), in close association with terms such as genetic diversity and apical membrane protein. Theme 2 focuses on the characterization, identification, and expression aspects of the vaccine's design. Theme 3 (purple cluster) targeted vaccine safety and efficacy through trials and the trial phases. RTS and PfSPz were two malaria vaccines associated with Theme 3. Theme 4 (blue cluster) primarily focuses on women, infants, and children in African countries such as Kenya, Ghana, and Burkina Faso. Theme 5 (blue-green cluster) concentrates mainly on travelers' prevention, including their knowledge, attitude, and practice. Theme 6 (red cluster) focuses on research and reviews on the disease in Africa.

## 4. Discussion

This study provides an overview of research output on the malaria vaccine through the bibliometric analysis of studies published from 2005 to 2022. The key findings of this analysis were discussed in comparison to a previous bibliometric analysis covering research from 1974 to 2004 [19]. Overall, 6457 documents were found in the current review, which was published in 160 journals in 189 countries/territories. This is in comparison to 2007 publications in 352 journals from 59 countries from 1974 to 2004 [19]. This demonstrates a notable expansion in the geographical diversity of malaria vaccine research over the past 1.5 decades.

Before 2012, the most cited research was on an experiment to produce an antimalarial precursor (artemisinic acid) to overcome multidrug resistance of *Plasmodium species* [27] while vaccines were still awaiting rigorous clinical trials. However, within the last decade (2012–2022), the most cited research was a clinical trial exploring the safety and efficacy of RTS, S malaria vaccine [28]. Both the current bibliometric analysis and the prior analysis show a steady increase in malaria vaccine publication output over the years, corresponding with the continued global efforts to produce effective malaria vaccines. However, there were not enough new directions for future research observed.

The period prior to 2005 to 2022 has seen a significant transition in malaria vaccine research from early experimental studies to more effective vaccine candidates. This can be seen in the success recorded on pre-erythrocytic vaccines targeting sporozoites, such as the RTS, S/AS01, the first malaria vaccine recommended by the WHO for use in sub-Saharan Africa (SSA) [29]. Accordingly, the analysis of keywords in this review indicates a high occurrence of RTS, S vaccine research, signifying the over three decades of RTS, S vaccine research and its associated challenges [30]. Recently, another vaccine, R21, was introduced with a promising result [15], leading to the approval of the vaccine by the WHO in October 2023 [31], indicating the continued progress recorded in malaria vaccine development. Over the periods of this study (2005–2022), the trends in malaria vaccine research cover broad areas of vaccine design, mainly focusing on the *Plasmodium* essential proteins (e.g., merozoite surface protein and apical membrane protein) and their characterization, vaccine targets (e.g., safety and efficacy), key population focus (e.g., infants and children), key countries in African (e.g., Kenya and Ghana), and contemporary issues like prevention of travelers. This indicates that malaria vaccine research from 2005 to 2022 has concentrated on development and testing and efforts to minimize international transmission from endemic to nonendemic countries. However, further research is needed to enhance vaccine efficacy, as the current vaccines have limited effectiveness.

Another trend identified in the current review is the link between malaria and other potential infectious disease vaccines such as typhoid, cholera, and rabies. This connection may be due to the similar signs and symptoms of these diseases with malaria. Additionally, all the diseases constitute a high burden of mortalities and morbidities, particularly in resource-poor settings in Africa and Asia [32, 33]. For instance, in 2021 alone, an estimated 108,859 cases of cholera and 3711 deaths (case fatality ratio of 3.4%) were reported in West Africa [33], necessitating the need for vaccines.

A further investigation into the keywords identified in this review indicates the different malaria parasites in relation to the vaccine research. *P. falciparum* has been considered the most prevalent parasite associated with malaria disease, accounting for 79.4 of malaria cases and 87.6% of mortalities in SSA [34]. The trend in *P. falciparum* is corroborated in the findings of this review, of which a vast majority of research output was attributed to falciparum and its essential proteins, var2csa protein and pfs25 protein. These proteins are crucial targets in developing malaria vaccines, representing the sexual stage surface antigen of *P. falciparum* [35, 36]. Another important species is the *P. vivax*, according to the findings of this review. While *P. falciparum* was associated with higher mortalities, *P. vivax* was demonstrated to be the most widespread species, presenting more severe and fatal illness [37]. Other malarial parasites, such as *P. knowelies*, were recently identified, particularly in South-East Asia, and could be associated with their unusual morphology coupled with the rare occurrence of cases [38].

Despite malaria being most prevalent in Africa, Kenya was the only African country among the Top 10 countries with the highest publication outputs and coauthorships with Western countries. In its 2021 report, the WHO African Region indicates a disproportionately high share of the global malaria burden in Africa, accounting for more than three-quarters of the malaria burden [3]. Similarly, children under the age of 5 accounted for about 80% of all malaria deaths in Africa, particularly in the SSA region [3]. The Top 3 countries (United States, United Kingdom, and Australia), accounting for about three-quarters of the publication outputs, conducted most of the studies in collaboration with African countries. Like the previous bibliometric analysis [19], the research output from the Top 3 countries (United States, United Kingdom, and Australia) contributed more than half of the current research, indicating the continued effort by research centers in these countries since 1974. This corresponds to the number of malaria research publications, of which the United States and the United Kingdom dominated over half of the output from 1970 to 2022 [6]. Nevertheless, the need for malaria research centers in Africa remains pertinent to promoting malaria vaccine research and successfully eradicating malaria globally.

Findings further indicate that neither SJR (weighted citation per output) nor the citescore (general citation per output) of the journals/sources of documents determined the number of publications. However, an open-access publication corresponded to the number of published documents, of which all the Top 10 journals except one published fully open access or a hybrid option (both open-access and traditional subscription options). Also, 8 of the 10 most cited papers in this study were published open access. While higher citations are popular as a motivation for publication in open-access journals [39], most of the malaria vaccine research received funding from central government and nongovernmental organizations such as the Bill and Melinda Gates Foundation; hence, it may have influenced the open-access publications. Additionally, the timely publication of open-access journals [40] may have influenced the publication of the malaria research as open access.

This bibliometric study covers a multidisciplinary approach, representing various research fields, including immunology, public health, epidemiology, and biochemistry. For instance, research on vaccine safety and efficacy primarily falls under clinical immunology and public health, as demonstrated in the RTS, S Clinical Trials Partnership study [41]. Exploring *Plasmodium* proteins such as merozoite surface protein and apical membrane antigen is rooted in biochemistry and molecular biology as reported in Sanders et al.'s study [42]. Epidemiological studies focusing on malaria's geographical spread and control measures highlight the importance of public health and preventive medicine. Furthermore, the coexistence of malaria with other infectious diseases like typhoid fever showcases the relevance of this research in global health and tropical medicine, as reported in the Sur et al. study [43]. This interdisciplinary representation underscores the complexity of malaria vaccine research and the necessity of collaborative efforts across various scientific domains to tackle this global health challenge.

Despite the strength of this review in presenting an overview of recent malaria vaccine research and building on a previous malaria vaccine bibliometric analysis [19], caution should be taken in interpreting the findings due to its limitations. First, like many other bibliometric analyses [22, 24], this study was based on a single database (Scopus); hence, research documents not indexed in Scopus could be missing. Second, the citation reports were also restricted to the Scopus database, which could be underestimated since citations by articles not indexed in Scopus were not counted. Third, the large number of documents identified and screened indicates the possibilities of both false positives and true negatives since the screening was primarily done using the Scopus interface futures. However, the validation process conducted was instrumental in significantly minimizing these errors.

## 5. Conclusion

This study shows a relative increase in malaria vaccine research and citations from 2005 to 2022. Key research themes within the study period include the development of vaccines targeting different aspects of *Plasmodium* species, focusing on efficacy/safety trials among children in African countries, and preventive measures for international travelers. Most of the research outputs were conducted by researchers in the United States, the United Kingdom, and Australia, with most of the research output in open-access journals. While *P. falciparum* and *P. vivax* remain the most researched malaria species, emerging research outputs were found in *P. knowlesi*. Further research in this area should focus on developing malaria vaccines for different malaria species and be aimed at improving the effectiveness of vaccines and the successful enrolment of vaccines in malaria-endemic countries.

## Figures and Tables

**Table 1:** Malaria vaccine research yearly publication and citation report.

**Year**	**Documents**	**Percentage (%)**	**Citation count**	**Average citation per document**
2022	441	6.8	1868	4.2
2021	468	7.3	4512	9.6
2020	397	6.2	6539	16.5
2019	373	5.8	6475	17.4
2018	370	5.7	8638	23.3
2017	399	6.2	11611	29.1
2016	387	6.0	14469	37.4
2015	380	5.9	12158	32.0
2014	352	5.5	12002	34.1
2013	369	5.7	13819	37.4
2012	345	5.3	13314	38.6
2011	365	5.7	14241	39.0
2010	385	6.0	17119	44.5
2009	300	4.7	15947	53.2
2008	299	4.6	17804	59.5
2007	326	5.1	15321	47.0
2006	252	3.9	13906	55.2
2005	249	3.9	14580	58.6
Total	6,457	100	214323	33.2

## Data Availability

The data that support the findings of this study are available on request from the corresponding author. The data are not publicly available due to privacy or ethical restrictions.

## Supporting Information

Supporting Information 1 Figure S1: malaria vaccine research co-occurrence of author's keywords.

Supporting Information 2 Figure S2: malaria vaccine research co-occurrence of index keywords.

Supporting Information 3 Figure S3: malaria vaccine research themes through density visualization.

## References

[1] Greenwood B. (2014). The contribution of vaccination to global health: past, present and future. *Philosophical Transactions of the Royal Society B: Biological Sciences*.

[2] Minor P. D. (2015). Live attenuated vaccines: historical successes and current challenges. *Virology*.

[3] World Health Organization (2023). *Malaria*.

[4] Shah N. K., Tyagi P., Sharma S. K. (2013). The impact of artemisinin combination therapy and long-lasting insecticidal nets on forest malaria incidence in tribal villages of India, 2006–2011. *PLoS One*.

[5] Chutiyami M., Saravanakumar P., Bello U. M. (2024). Malaria vaccine efficacy, safety, and community perception in Africa: a scoping review of recent empirical studies. *Infection*.

[6] Aydemir S., Diril H., Alkan S., Barlık F., Ekici A. (2024). A bibliometric analysis study of global academic articles on malaria and contribution of Türkiye. *Journal of Health Science*.

[7] Sheehy S. H., Douglas A. D., Draper S. J. (2013). Challenges of assessing the clinical efficacy of asexual blood-stage *Plasmodium falciparum* malaria vaccines. *Human vaccines & immunotherapeutics*.

[8] Tsoumani M. E., Voyiatzaki C., Efstathiou A. (2023). Malaria vaccines: from the past towards the mRNA vaccine era. *Vaccines*.

[9] Vaughan A. M., Wang R., Kappe S. H. I. (2010). Genetically engineered, attenuated whole-cell vaccine approaches for malaria. *Human Vaccines*.

[10] Yusuf Y., Yoshii T., Iyori M. (2019). A viral-vectored multi-stage malaria vaccine regimen with protective and transmission-blocking efficacies. *Frontiers in immunology*.

[11] Makoni M. (2023). mRNA vaccine against malaria effective in preclinical model. *The Lancet Microbe*.

[12] Oneko M., Steinhardt L. C., Yego R. (2021). Safety, immunogenicity and efficacy of PfSPZ Vaccine against malaria in infants in western Kenya: a double-blind, randomized, placebo-controlled phase 2 trial. *Nature Medicine*.

[13] Sagara I., Zongo I., Cairns M. (2022). The anti-circumsporozoite antibody response of children to seasonal vaccination with the RTS,S/AS01E Malaria vaccine. *Clinical Infectious Diseases*.

[14] The RTS,S Clinical Trials Partnership (2012). A phase 3 trial of RTS, S/AS01 malaria vaccine in African infants. *New England Journal of Medicine*.

[15] Datoo M. S., Natama H. M., Somé A. (2022). Efficacy and immunogenicity of R21/matrix-M vaccine against clinical malaria after 2 years’ follow-up in children in Burkina Faso: a phase 1/2b randomised controlled trial. *The Lancet Infectious Diseases*.

[16] Cairns M., Barry A., Zongo I. (2022). The duration of protection against clinical malaria provided by the combination of seasonal RTS, S/AS01E vaccination and seasonal malaria chemoprevention versus either intervention given alone. *BMC Medicine*.

[17] Chandramohan D., Zongo I., Sagara I. (2021). Seasonal malaria vaccination with or without seasonal malaria chemoprevention. *New England Journal of Medicine*.

[18] Datoo M. S., Natama M. H., Somé A. (2021). Efficacy of a low-dose candidate malaria vaccine, R21 in adjuvant matrix-M, with seasonal administration to children in Burkina Faso: a randomised controlled trial. *The Lancet*.

[19] Garg K. C., Kumar S., Madhavi Y., Bahl M. (2009). Bibliometrics of global malaria vaccine research. *Health Information & Libraries Journal*.

[20] Donthu N., Kumar S., Mukherjee D., Pandey N., Lim W. M. (2021). How to conduct a bibliometric analysis: an overview and guidelines. *Journal of Business Research*.

[21] de Oliveira O. J., da Silva F. F., Juliani F., LCFM B., Nunhes T. V. (2019). Bibliometric method for mapping the state-of-the-art and identifying research gaps and trends in literature: an essential instrument to support the development of scientific projects. *Scientometrics recent advances*.

[22] Sweileh W. M. (2020). Bibliometric analysis of global scientific literature on vaccine hesitancy in peer-reviewed journals (1990-2019). *BMC Public Health*.

[23] Xu Z., Qu H., Ren Y. Y. (2021). Update on the COVID-19 vaccine research trends: a bibliometric analysis. *Infection and Drug Resistance*.

[24] Sweileh W. M. (2020). Bibliometric analysis of peer-reviewed literature on climate change and human health with an emphasis on infectious diseases. *Globalization and Health*.

[25] Pranckutė R. (2021). Web of Science (WoS) and Scopus: The titans of bibliographic information in today’s academic world. *Publications*.

[26] Harzing A. W., Alakangas S. (2016). Google Scholar, Scopus and the Web of Science: a longitudinal and cross-disciplinary comparison. *Scientometrics*.

[27] Ro D. K., Paradise E. M., Ouellet M. (2006). Production of the antimalarial drug precursor artemisinic acid in engineered yeast. *Nature*.

[28] RTS,S Clinical Trials Partnership (2015). Efficacy and safety of RTS, S/AS01 malaria vaccine with or without a booster dose in infants and children in Africa: final results of a phase 3, individually randomised, controlled trial. *The Lancet*.

[29] World Health Organization (2022). *Malaria vaccine: WHO position paper - March 2022. Weekly Epidemiological Record*.

[30] World Health Organization (2020). World malaria report: 20 years of global progress and challenges. https://www.who.int/publications/i/item/9789240015791.

[31] World Health Organization (2023). *WHO recommends R21/matrix-M vaccine for malaria prevention in updated advice on immunization*.

[32] Jiang L., Huang T. (2019). Comparison of the epidemiological aspects of acute infectious diseases between foreign and native imported cases in the border counties of Southwest China, 2008–2017. *Epidemiology and Infection*.

[33] Sodjinou V. D., Talisuna A., Braka F. (2022). The 2021 cholera outbreak in West Africa: epidemiology and public health implications. *Archives of Clinical and Biomedical Research*.

[34] Weiss D. J., Lucas T. C. D., Nguyen M. (2019). Mapping the global prevalence, incidence, and mortality of *Plasmodium falciparum*, 2000–17: a spatial and temporal modelling study. *The Lancet*.

[35] Sookpongthai P., Utayopas K., Sitthiyotha T. (2021). Global diversity of the gene encoding the Pfs 25 protein—a *Plasmodium falciparum* transmission-blocking vaccine candidate. *Parasites & Vectors*.

[36] Renn J. P., Doritchamou J. Y. A., Tentokam B. C. N. (2021). Allelic variants of full-length VAR2CSA, the placental malaria vaccine candidate, differ in antigenicity and receptor binding affinity. *Communications Biology*.

[37] Menkin-Smith L., Winders W. T. (2023). *Plasmodium vivax Malaria*.

[38] Aftab H., Kemp M., Stensvold C. R. (2023). First molecular documented case of a rarely reported parasite: *Plasmodium knowlesi* infection in Denmark in a traveller returning from Malaysian Borneo. *Travel Medicine and Infectious Disease*.

[39] Donovan J. M., Watson C. A. (2011). Citation advantage of open access legal scholarship. *Law Library Journal*.

[40] Solomon D. J., Björk B. C. (2012). Publication fees in open access publishing: sources of funding and factors influencing choice of journal. *Journal of the American Society for Information Science and Technology*.

[41] RTS,S Clinical Trials Partnership (2014). Efficacy and safety of the RTS, S/AS01 malaria vaccine during 18 months after vaccination: a phase 3 randomized, controlled trial in children and young infants at 11 African sites. *PLoS Medicine*.

[42] Sanders P. R., Gilson P. R., Cantin G. T. (2005). Distinct protein classes including novel merozoite surface antigens in raft-like membranes of *Plasmodium falciparum*. *Journal of Biological Chemistry*.

[43] Sur D., von Seidlein L., Manna B. (2006). The malaria and typhoid fever burden in the slums of Kolkata, India: data from a prospective community-based study. *Transactions of the Royal Society of Tropical Medicine and Hygiene*.

