# Supplementary figures and images for: Recent Trends in Malaria Vaccine Research Globally: A Bibliometric Analysis From 2005 to 2022
Source: J Parasitol Res. 2024 Oct 24;2024:8201097. doi: 10.1155/2024/8201097 (PMC11527547; doi:10.1155/2024/8201097)

Supplemental Figure 1: Malaria Vaccine Research Co-occurrence of Author's Keywords

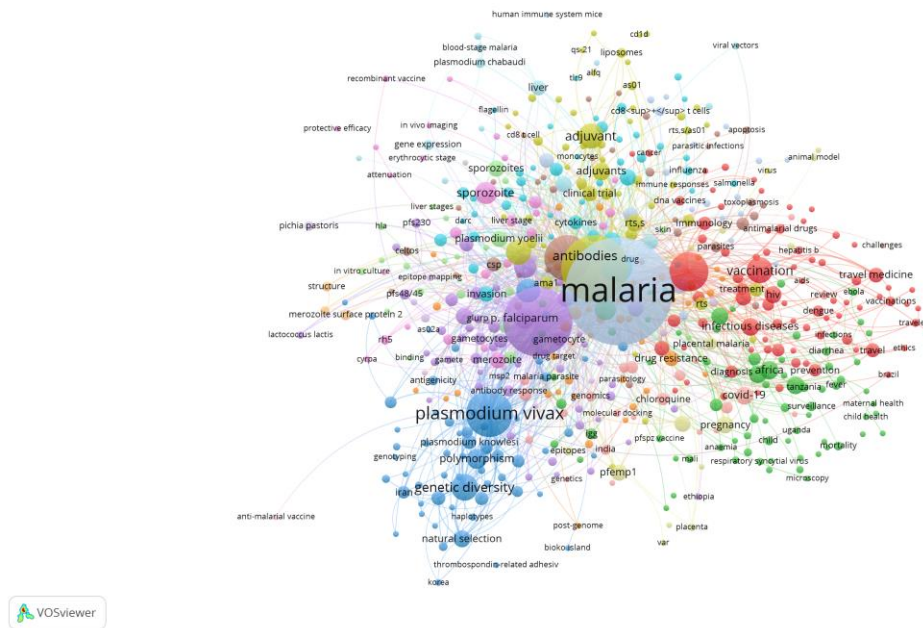

Supplement: Supporting Information 1 — Figure S1: malaria vaccine research co-occurrence of author's keywords. [file 8201097.f1.pdf]

Supplemental Figure 2: Malaria Vaccine Research Co-occurrence of Index Keywords

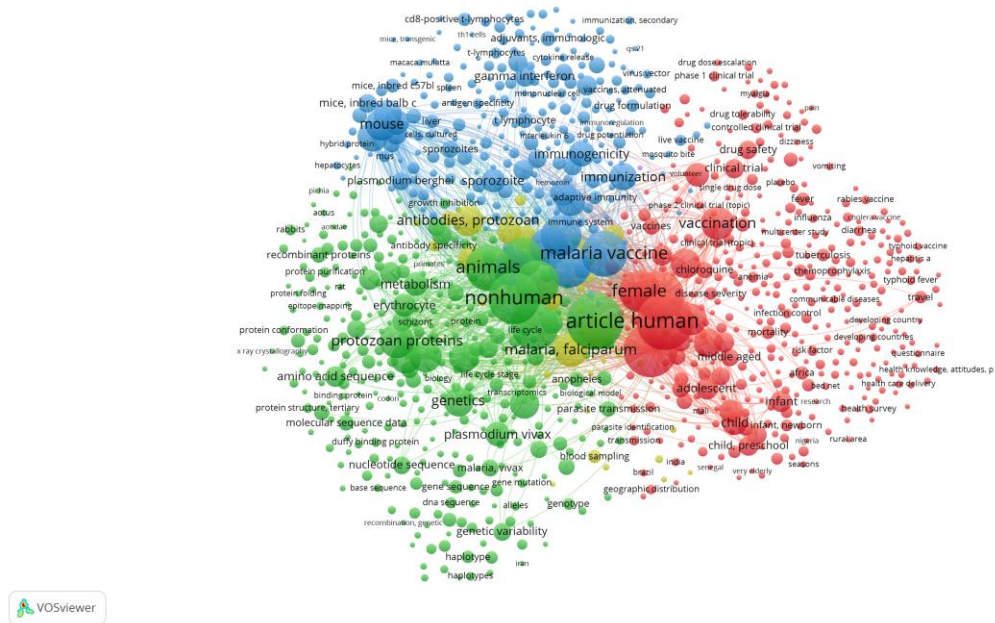

Supplement: Supporting Information 2 — Figure S2: malaria vaccine research co-occurrence of index keywords. [file 8201097.f2.pdf]

Supplemental Figure 3: Malaria Vaccine Research Themes through Density Visualization

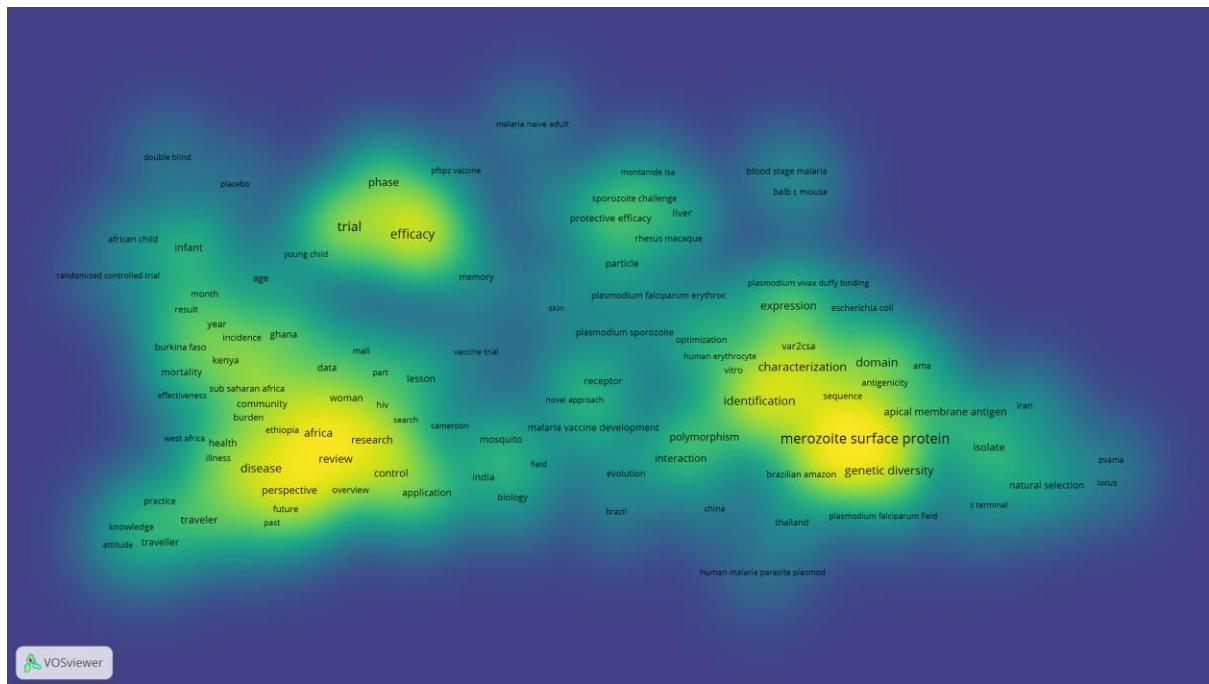

Supplement: Supporting Information 3 — Figure S3: malaria vaccine research themes through density visualization. [file 8201097.f3.pdf]
